# Regulation of differentiation flux by Notch signalling influences the number of dopaminergic neurons in the adult brain

**DOI:** 10.1242/bio.013383

**Published:** 2016-02-24

**Authors:** Niurka Trujillo-Paredes, Concepción Valencia, Gilda Guerrero-Flores, Dulce-María Arzate, José-Manuel Baizabal, Magdalena Guerra-Crespo, Ayari Fuentes-Hernández, Iván Zea-Armenta, Luis Covarrubias

**Affiliations:** 1 Departamento de Genética y Fisiología Molecular, Instituto de Biotecnología, Cuernavaca, Morelos 62210, México; 2 Departamento de Neuropatología Molecular, Instituto de Fisiología Celular, Coyoacán, Ciudad de México 04510, México; 3 Centro de Ciencias Genómicas, Universidad Nacional Autónoma de México, Cuernavaca, Morelos 62210, México

**Keywords:** Delta-like 1, Notch, Neurogenesis, Dopaminergic neurons

## Abstract

Notch signalling is a well-established pathway that regulates neurogenesis. However, little is known about the role of Notch signalling in specific neuronal differentiation. Using *Dll1* null mice, we found that Notch signalling has no function in the specification of mesencephalic dopaminergic neural precursor cells (NPCs), but plays an important role in regulating their expansion and differentiation into neurons. Premature neuronal differentiation was observed in mesencephalons of *Dll1*-deficient mice or after treatment with a Notch signalling inhibitor. Coupling between neurogenesis and dopaminergic differentiation was indicated from the coincident emergence of neuronal and dopaminergic markers. Early in differentiation, decreasing Notch signalling caused a reduction in NPCs and an increase in dopaminergic neurons in association with dynamic changes in the proportion of sequentially-linked dopaminergic NPCs (Msx1/2+, Ngn2+, Nurr1+). These effects in differentiation caused a significant reduction in the number of dopaminergic neurons produced. Accordingly, *Dll1* haploinsufficient adult mice, in comparison with their wild-type littermates, have a consistent reduction in neuronal density that was particularly evident in the substantia nigra pars compacta. Our results are in agreement with a mathematical model based on a Dll1-mediated regulatory feedback loop between early progenitors and their dividing precursors that controls the emergence and number of dopaminergic neurons.

## Abstract (precis)

**Summary:** The early emergence of dopaminergic neurons under reduced Notch signalling results from a change in the differentiation flux that defines the final number of neurons produced.

## INTRODUCTION

At the cellular level, early embryogenesis involves stem and progenitor cell proliferation followed by their exit from the cell cycle and concurrent differentiation into specific cell types. In this context, the size and shape of the nervous system largely depend on the number of times that a neural stem cell (NSC) or its progeny re-enters the cell cycle. Importantly, timing of cell differentiation may also influence cell fate choice, given the fact that NSCs appear to change their potential over time throughout development (Bassett and Wallace, 2012; Okano and Temple, 2009). In particular, it has been shown that specific cortical neurons arise at a different developmental time, suggesting that specification is associated with the time of birth (Okano and Temple, 2009; Shen et al., 2006). Nonetheless, it is still unclear how cell differentiation timing influences cell fate choice and the histogenesis of specific brain regions.

The evolutionarily conserved Notch signalling pathway mediates cell-cell interactions that regulate the process of differentiation of neighbouring cells, providing a mechanism for consistent cell fate determination and patterning in time and space of highly organized tissues (Louvi and Artavanis-Tsakonas, 2006; Pierfelice et al., 2011). Notch is a family of transmembrane receptors that are activated by transmembrane ligands such as Delta-like (Dll1, Dll3 and Dll4) and Jagged (Jag1 and Jag2) in mammals. Upon activation, the Notch intracellular domain (NICD) is released and translocated to the nucleus, where forms a complex with the DNA-binding protein RBPj (Pierfelice et al., 2011). In the CNS of mammals, the NICD-RBPj complex induces the expression of *Hes1* and *Hes5*, genes encoding basic helix-loop-helix transcription factors that, in turn, can repress the expression of pro-neural genes (including Notch ligand genes), thereby inhibiting neuronal differentiation and maintaining the pool of neural precursor cells (NPC) (Ohtsuka et al., 1999). Blocking this pathway at different levels causes premature differentiation of NPC resulting in reduction in the number and spectrum of neuron types (Hatakeyama et al., 2004; Louvi and Artavanis-Tsakonas, 2006; Ohtsuka et al., 1999). Thus, Notch signalling appears to be an essential component of the mechanisms that lead to the production of the neuronal diversity characteristic of the brain starting from apparently equivalent NSCs.

Despite the above, very little is known about the role of Notch signalling in the generation of specific brain regions and/or neuron types. In the developing midbrain, *Notch1*,* 2*,* 3*,* Dll1* and *Jag1* are expressed in the ventricular zone (Lindsell et al., 1996). *Notch1* and *Notch2* in rodents are essential genes (Conlon et al., 1995; Swiatek et al., 1994) but, while *Notch2* appears mainly involved in diencephalon and mesencephalon roof plate development (Kadokawa and Marunouchi, 2002), conditional *Notch1* deletion along the midbrain-hindbrain region results in the premature onset of neurogenesis (Lütolf et al., 2002). *Dll1* and *Dll4* are also essential genes in early mouse development (Duarte et al., 2004; Hrabĕ de Angelis et al., 1997); *Dll1*, in addition to be transiently expressed during gastrulation and early organogenesis, during CNS development is expressed in most of the neural tube (Bettenhausen et al., 1995). Interestingly, in contrast with other brain regions, the mesencephalic floor plate (FP) expresses *Dll1* (Ono et al., 2007), which associates with the peculiar neurogenic activity of this region (Joksimovic et al., 2009; Ono et al., 2007). Gene expression patterns and NPC differentiation potential of cells in the mesencephalic ventral midline (Lin et al., 2009; Ono et al., 2007) as well as fate mapping experiments (Kittappa et al., 2007) indicate that mesencephalic dopaminergic neurons originate from precursors within the FP. Therefore, *Dll1* may play a role in the positioning, maintenance, and patterning of dopaminergic neurons and their NPCs.

Dopaminergic differentiation is characterized by the sequential expression of genes encoding certain transcription factors (e.g. En2, Otx2, Foxa2, Lmx1a, Msx1, Ngn2, Nurr1, Pitx3), which are downstream targets of extrinsic signals such as Shh, Fgf8 and Wnt1 (for a review see: Abeliovich and Hammond, 2007; Ang, 2006; Guerrero-Flores and Covarrubias, 2011; Hegarty et al., 2013). These transcription factors regulate the transition between different cell populations along the ventricular-alar axis of the developing ventral mesencephalon. Interestingly, Ngn2 and Mash1, recognized as proneural transcription factors, control the expression of *Dll1* and, in consequence, also of some genes associated with Notch signalling, such as *Hes5* (Castro et al., 2006; Kele et al., 2006).

Regulation of expression of  Notch signalling genes has been studied in association with mesencephalic dopaminergic differentiation (Castro et al., 2006; Deng et al., 2011; Kele et al., 2006; Ono et al., 2007); however, in contrast, little is known about how Notch signalling regulates dopaminergic differentiation. In this study, we investigated the function of Notch signalling in the control of dopaminergic neurogenesis and the number of dopaminergic neurons produced.

## RESULTS AND DISCUSION

### Dll1 and Hes5 are key mediators of Notch signalling in the mesencephalic dopaminergic niche

**Fig. 1.**
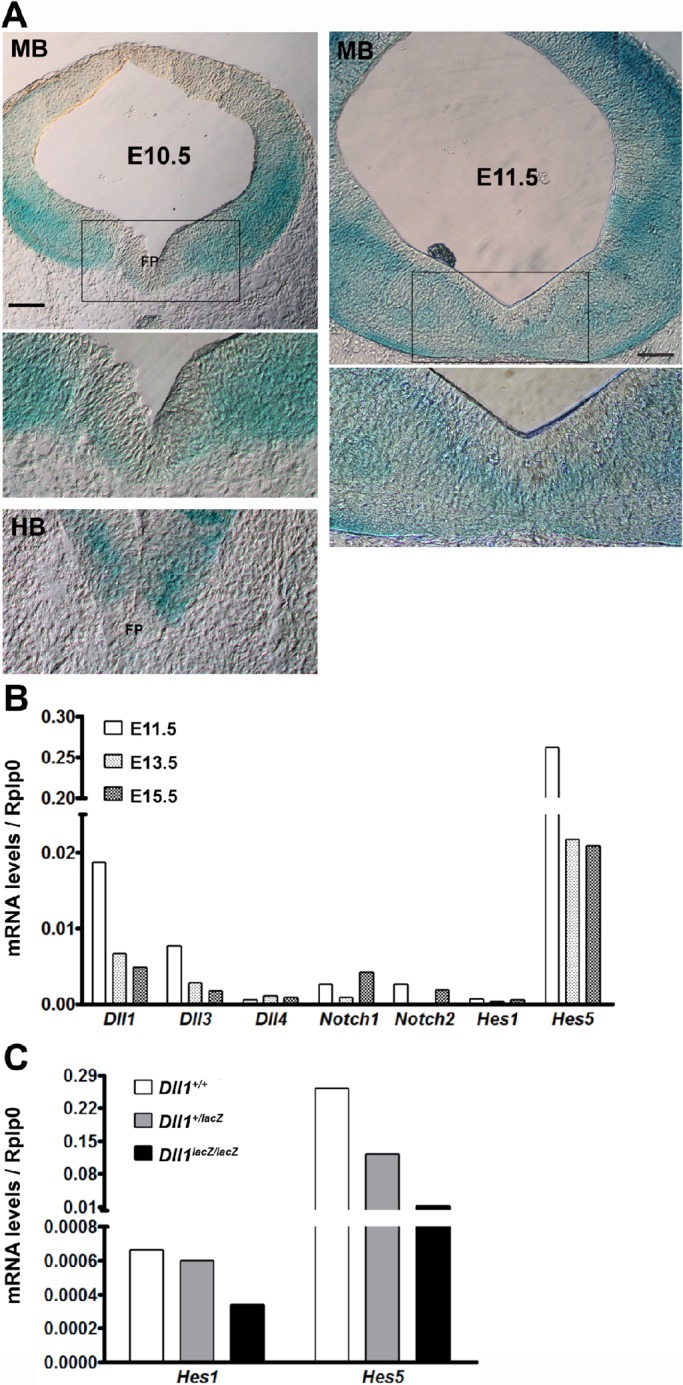
**Dll1-Notch signalling in the mesencephalic dopaminergic niche.** (A) Midbrains (MB) or hindbrains (HB) from *Dll1^+/lacZ^* mouse embryos at the stage indicated were stained for the *lacZ* reporter and slices from them are shown. FP, floor plate. Scale bar is 100 µm. (B) Total RNA was extracted from wild-type mouse embryos (pool of 10) at different stages (E11.5, E13.5 and E15.5) and the expression level of the genes indicated determined by RT-qPCR. Note that *Dll1* and *Dll3* followed a similar expression pattern that closely correlated with the pattern of *Hes5*. (C) *Hes1* and *Hes5* expression pattern was determined in E11.5 embryos with the genotype indicated. Note that *Hes5* markedly decreased (near 30-fold) in the absence of Dll1.

*Dll1* transcript distribution in the developing mesencephalon has been previously determined by *in situ* hybridization; however, probably due to the quantitative limitations of this technique, the expression pattern has not been well defined showing scattered distribution with an apparent higher number of positive cells towards the subventricular area (Deng et al., 2011; Kele et al., 2006; Lahti et al., 2011). Here, we estimated *Dll1* transcript distribution by *in situ* determination of *lacZ* activity in *Dll1^+/lacZ^* mouse embryos, particularly at the initiation of dopaminergic differentiation. In the mesencephalon of embryonic day (E)10.5 and E11.5 embryos, *Dll1* expression occurred mainly in the subventricular area with the highest levels found in the ventral half. Particularly in the dopaminergic niche at E10.5, the floor plate showed a thin layer of *lacZ*-stained cells just below the ventricular epithelium, which contrast with the pattern in the hindbrain (Fig. 1A). In E11.5 mesencephalons, a wider subventricular area of *Dll1* expression was found around the ventral midline, corresponding to the location of intermediate progenitors, (Fig. 1A). At this latter stage, the ventral mesencephalon contained higher mRNA levels of *Dll1* than of *Dll3* or *Dll4*, whereas those of *Notch1* and *Notch2* were similar (Fig. 1B). In order to determine whether Dll1 is responsible for most Notch signalling occurring in the floor plate of mesencephalon in association with dopaminergic neuron differentiation, we compared the expression levels of two Notch effector genes, *Hes1* and *Hes5*, in the ventral mesencephalon of E11.5 embryos lacking Dll1. Both *Hes1* and *Hes5* were expressed in wild-type samples, but the latter was apparently more than 100-fold more abundant than the former (Fig. 1B). Interestingly, the complete absence of Dll1 levels caused a corresponding near 30-fold reduction in *Hes5* expression, whereas *Hes1* expression was only partially (about half) affected (Fig. 1C). In agreement with this conclusion, developmental downregulation of *Dll1* expression from E11.5 to E15.5 was best correlated with the expression level of *Hes5* (Fig. 1B). Therefore, Dll1 and Hes5 are the major upstream and downstream mediators, respectively, of Notch signalling in the developing ventral mesencephalon.

### Reduced Notch signalling alters the number of dopaminergic precursor cells without affecting their specification

**Fig. 2.**
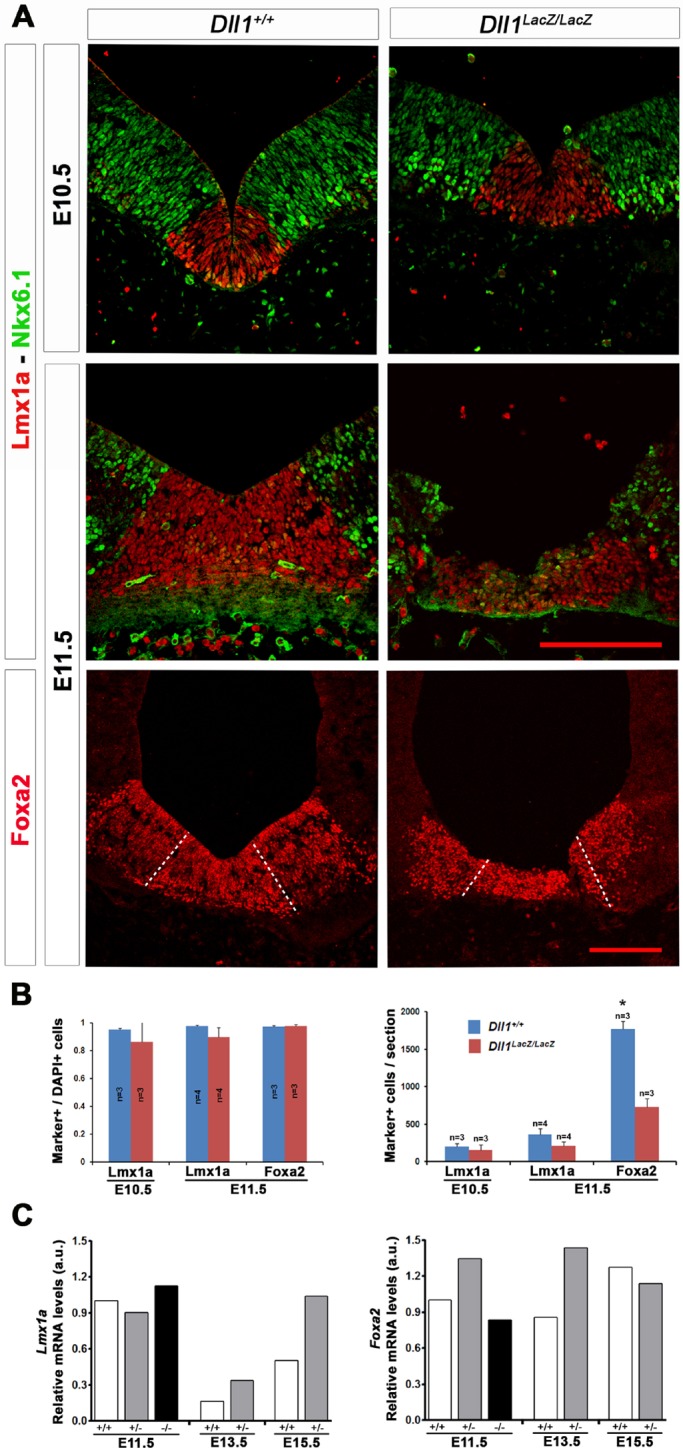
**Expression pattern of specification marker genes in the absence of Dll1.** (A) The presence of Lmx1a, Foxa2 and Nkx6.1 was determined in sections of midbrain from E10.5 and E11.5 *Dll1^+/+^* and *DII1^lacZ/lacZ^* mouse embryos. Scale bars are 200 µm. (B) The proportion of cells within the domain expressing each specific gene was not affected by the absence of Dll1, but there was a marked reduction in the number of Lmx1a+ and Foxa2+ cells in midbrains of *Dll* null mice at E11.5. Data represented as percentage of the total cell number (DAPI+ cells)±s.d.; *n*=5; *P<0.05. (C) *Lmx1a* and *Foxa2* mRNA expression levels were consistent with the previous observations (i.e. same proportion of cells with the corresponding marker).

In order to determine whether Notch signalling has any role in maintaining the organization of the dopaminergic domain in the ventral mesencephalon, we compared the distribution pattern of Lmx1a and Foxa2 in *Dll1^+/+^* and *Dll1^lacZ/lacZ^* embryos. We also determined the distribution pattern of Nkx6.1; the gene encoding this transcription factor is expressed lateral to the *Lmx1a* expression domain and is repressed in dopaminergic NPCs after proper specification (Andersson et al., 2006b; Nakatani et al., 2010). Specification of dopaminergic NPC occurs between E9-E10 and neuronal differentiation markers start to be detected from E11 with a peak between E12 and E13 (Ang, 2006). *Dll1* null mouse embryos die by E12 (Hrabĕ de Angelis et al., 1997, and our own observations), therefore, we limited the *in vivo* studies up to E11.5, stage at which most embryos are still alive though abnormalities were evident (Fig. S1). As shown in Fig. 2, the distribution pattern of Lmx1a, Foxa2 and Nkx6.1 is similar in midbrains of *Dll1^+/+^* and *Dll1^lacZ/lacZ^* embryos at E10.5 and E11.5; however, at E11.5, although restricted distribution of each protein was still observed, the tissue seemed disorganized and fragile with fewer cells Lmx1a+ and Foxa2+ in mesencephalons of mutant than in those of wild-type embryos (Fig. 2B). Note, that within each specific expression domain, a high proportion of cells contained the corresponding marker (Fig. 2B), indicating that the decrease in number of presumably specified cells in mesencephalons of mice lacking Dll1 is not due to a failure in maintaining the mesencephalic dopaminergic fate. In agreement with these observations, *Lmx1a* and *Foxa2* mRNA levels were similar in ventral mesencephalons of *Dll1^+/+^* and *Dll1^lacZ/lacZ^* embryos at E11.5 (Fig. 2C). Interestingly, consistent *Lmx1a* up-regulation was observed in *Dll1^+/lacZ^* embryos between E13.5 and E15.5 (Fig. 2C). Since *Lmx1a* mRNA levels increased after the rapid decrease in association with specification and differentiation, this latter effect was likely related to a Lmx1a function in neuronal maturation. Although less conspicuous, *Foxa2* expression at E13.5 showed a similar correlation (Fig. 2C; see also observations after Notch signalling inhibition in Fig. S2). Therefore, alterations in Notch signalling do not appear to affect dopaminergic niche specification.

### The poor expansion of dopaminergic NPCs lacking Dll1 correlates with the premature detection of neuronal markers

There was no evident increase in cell death around the midline in mutant embryos at E10.5 and, at E11.5 (Fig. S3A, left panels). In contrast, the thinner neural tube of mesencephalons of E11.5 *Dll1* null embryos correlated with a marked reduction in the number of cells that incorporated BrdU in comparison with the number observed in mesencephalons of wild-type embryos (Fig. S3A, right panels and Fig. S3B). These data suggest that, in the absence Dll1, the pool of ventral NPCs lining the ventricular zone of mesencephalon decreased due to a diminished capacity to proliferate and/or to a premature differentiation.

**Fig. 3.**
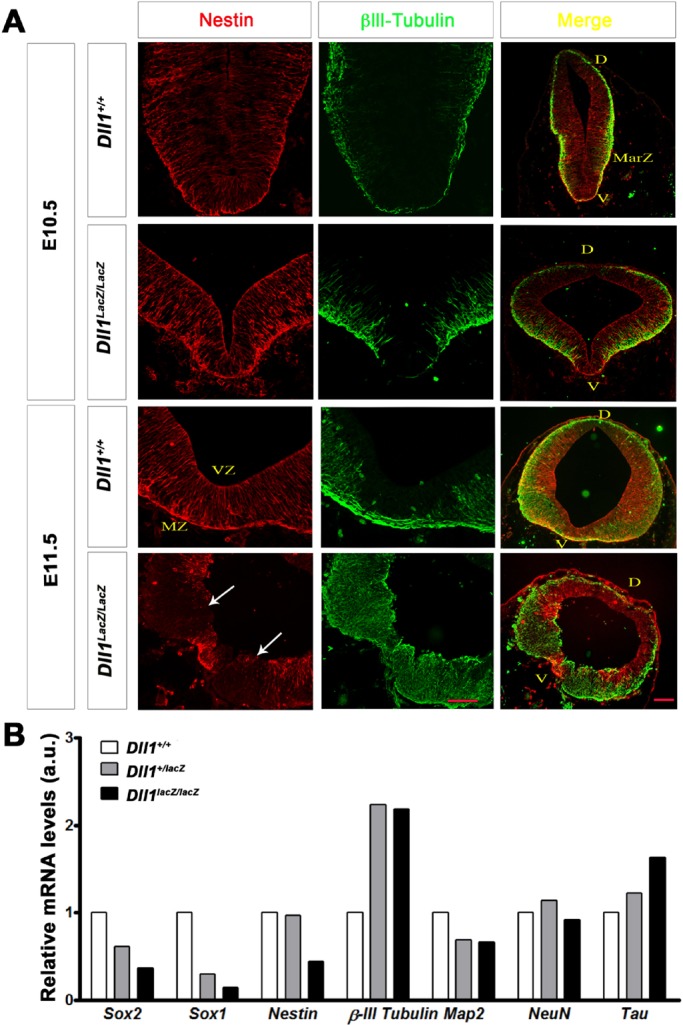
**Neuronal differentiation in developing midbrains in the presence or absence of DII1.** (A) At E10.5, Nestin (red) cover most of the midbrain neuroepithelium and, although a significant amount of βIII-tubulin started to be detected (green) in the marginal zone of samples from mutant embryos, this was not evident in the most ventral (V) or dorsal (D) area (upper panels); note that the lumen of midbrains from *Dll^lacZ/lacZ^* embryos expanded earlier than that of midbrains from wild-type embryos. At E11.5, along with a reduction in Nestin+ cells, abundant βIII-tubulin was detected in the ventral (V) area of midbrains from *Dll^lacZ/lacZ^* embryos, which was distributed along the whole neuroepithelium thickness; a number of Nestin+ cells remained in the midline of most cases analysed. Arrows indicate the limits of the floor plate in a mutant sample. MarZ, marginal zone; VZ, ventricular zone; MZ, mantle zone. Scale bars are 200 µm. (B) The observations in A were confirmed by determining the expression levels of neural precursor (*Sox1*, *Sox2*, *Nestin*) and neuronal (*βIII-tubulin*, *Map2*, *NeuN*, *Tau*) genes in ventral midbrains of embryos with the Dll1 genotype indicated. Note that there was a no marked alteration in the expression of genes associated with mature neurons (*Map2*, *NeuN*, *Tau*).

The typical radial distribution of NPCs (Nestin+ cells) was observed in *Dll1* deficient midbrains at E10.5 but was altered by E11.5 (Fig. 3A). Interestingly, the abundance and distribution of the immature neuronal marker βIII-tubulin suggest that neurogenesis is at a more advance stage in the mesencephalons of *Dll1^lacZ/lacZ^* embryos since E10.5, and became more evident by E11.5 in comparison with wild-type mesencephalons at an equivalent developmental stage (Fig. 3A). Marked reduction in Nestin+ cells and the extension of those βIII-tubulin+ to the ventricular zone was observed at E11.5 in the ventral region of mesencephalons lacking Dll1 (Fig. 3A). Of note was a pool of Nestin+ cells that were commonly detected around the midline in mutant mice; the identity of these cells remains to be determined (see Concluding remarks). Positive cells for NeuN, a mature neuronal marker, were not detected in the dopaminergic niche, even under the precocious differentiation observed in embryos lacking Dll1 (data not shown and see below). An expression analysis of neurogenic genes in the ventral mesencephalon of *Dll1^+/+^*, *Dll1^+/lacZ^* and *Dll1^lacZ/lacZ^* embryos at E11.5 also supports premature neuronal differentiation with little or no marked alterations in the expression of genes associated with mature neurons (Fig. 3B). Of note was that, with the exception of *Nestin* expression, the gene expression levels observed in ventral mesencephalons from *Dll1^lacZ/lacZ^* embryos were similar to those in samples from *Dll1^+/lacZ^* embryos.

**Fig. 4.**
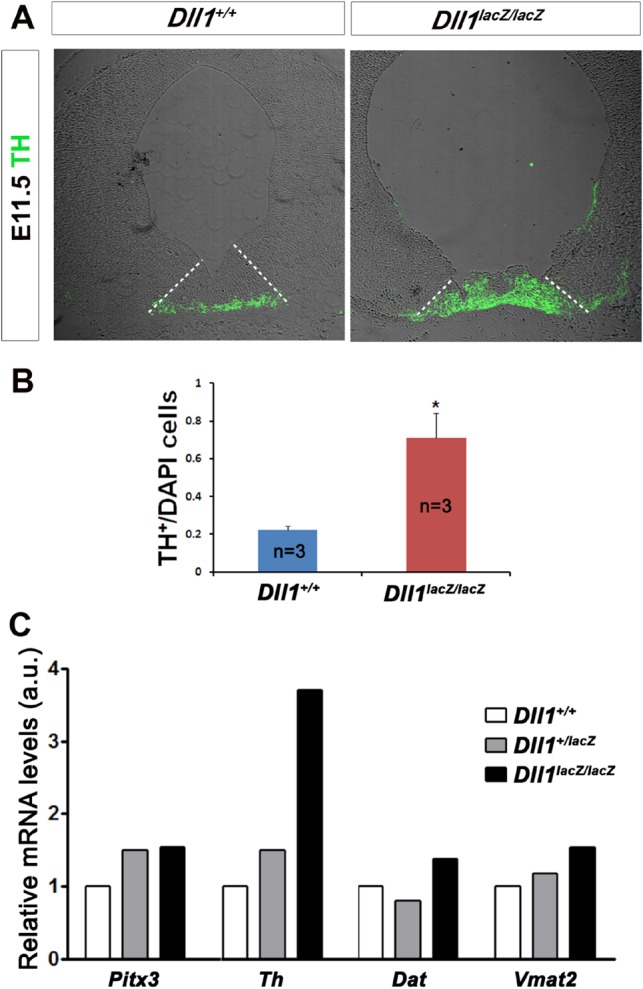
**Dopaminergic differentiation in developing midbrains in the presence or absence of DII1.** Dopaminergic differentiation was determined by (A,B) detecting the cells containing tyrosine hydroxylase (Th), the limiting enzyme in dopamine synthesis, or the expression level of its gene (C) in ventral midbrains of E11.5 embryos with the genotype indicated. A significantly larger number of putative dopaminergic neurons were detected in samples from *Dll^lacZ/lacZ^* in comparison with wild-type embryos (A,B), which positively correlated with the expression levels of *Th* but not of genes that are expressed in mature dopaminergic neurons (C). Scale bar is 100 µm. White dotted lines indicate the limits of the dopaminergic domain (estimated by the area expressing Lmx1a). Data represented as percentage of the total cell number (DAPI+ cells)±s.d.; *n*=5; *P<0.05.

Positive cells for Tyrosine hydroxylase (Th), a limiting enzyme in the synthesis of dopamine and one of the earliest markers of dopaminergic neurons, were detected at about the same time in wild-type and mutant tissues (E11.5), but their distribution in the latter samples resembled a more advanced developmental stage (i.e. E12.5) (Fig. 4A). In addition, cell quantification revealed a significant difference in the proportion of Th+ cells between mutant and control midbrain tissues at E11.5 (Fig. 4B). In agreement with the premature emergence of Th in mutant mice, *Th* mRNA levels were elevated in ventral mesencephalon of *Dll1^lacZ/lacZ^* embryos (Fig. 4C), in close similarity with the increase in βIII-tubulin; a marginal increase was detected in heterozygous embryos. The mRNA levels of *Vmat* and *Dat*, markers of mature dopaminergic neurons, did not markedly change between the different genotypes at E11.5, probably because the analysis was done at an early stage of differentiation.

### Mesencephalic explant cultures recapitulate the effects of Notch signalling deficiency on dopaminergic neuronal differentiation

It was not unexpected to find that the premature neuronal differentiation described above at E11.5 was not reflected in the expression of genes encoding proteins associated with mature neurons (e.g. NeuN). Since mesencephalic dopaminergic differentiation was not completed before *Dll^lacZ/lacZ^* embryos die, we analysed the differentiation potential of mesencephalic NPCs in culture.

**Fig. 5.**
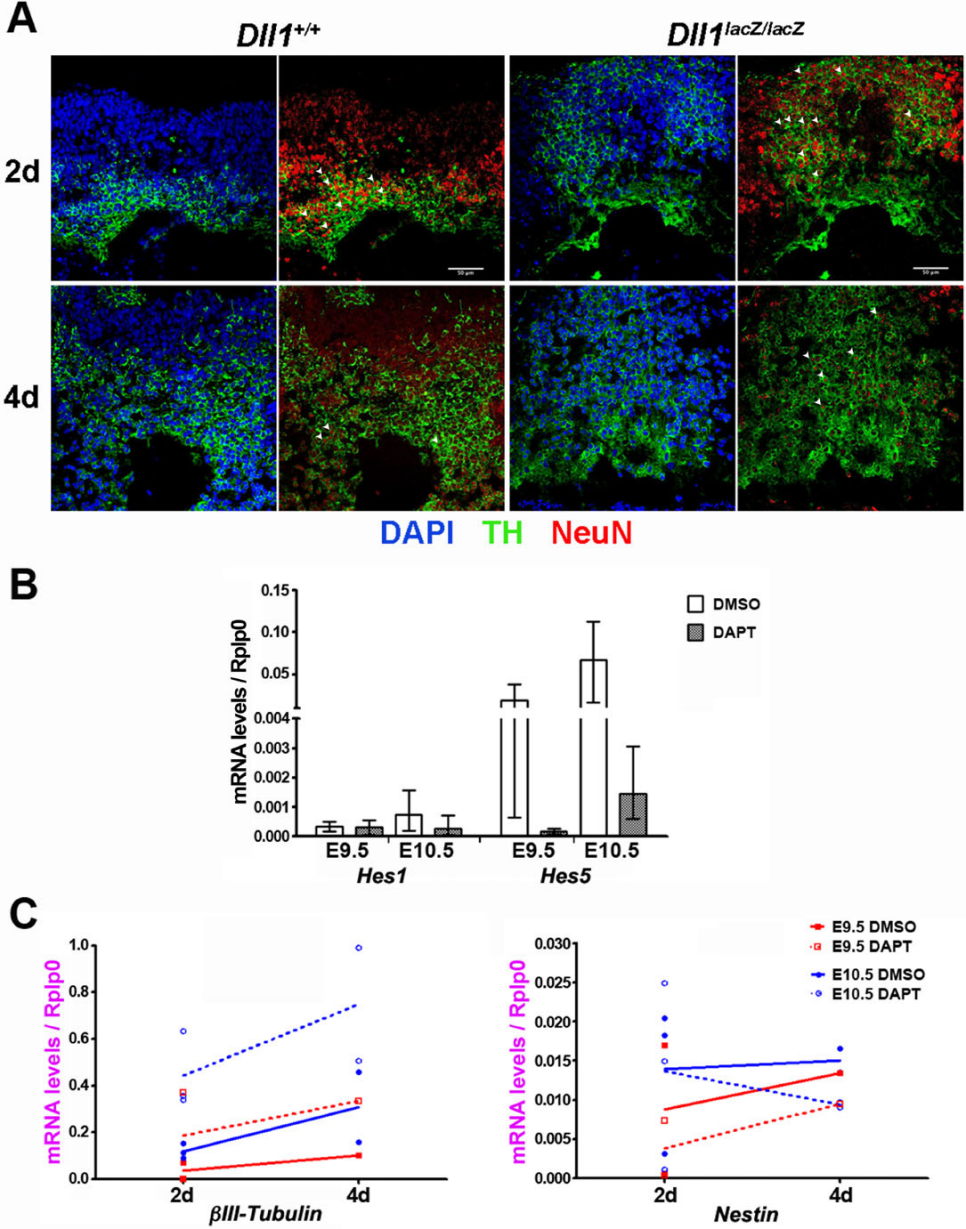
**Effect of Notch signalling on neuronal dopaminergic differentiation in explant cultures of developing mesencephalons.** Neuronal dopaminergic differentiation was determined by detecting the number of cells containing the mature neuronal marker NeuN (some indicated with arrowheads) and Th (A) or the mRNA level of neural precursor (Nestin) or neuronal (βIII-tubulin) markers (C). Mesencephalons with no or low Notch signalling were those derived from *Dll^lacZ/lacZ^* embryos (A) or treated with the DAPT Notch inhibitor for 2 days (2 d) or 4 days (4 d) (B,C). Note that the treatment (2d) of E9.5 and E10.5 explant cultures with the Notch inhibitor DAPT markedly reduced the expression of *Hes5* with a minimum effect on *Hes1* (B), in agreement with the effect determined in embryos lacking *Dll1*. Delayed DAPT effect (2 days vs 4 days) on *Nestin* expression in culture of E10.5 mesencephalons could be related to the ventricular NPC population that is not committed to become neurons. Scale bars are 100 µm. Data in B represented as average±highest/lowest values.

Explant cultures embedded in collagen allow dopaminergic differentiation to a stage resembling the distribution and number of dopaminergic neurons present in the mesencephalon of E14-E15 embryos (Baizabal and Covarrubias, 2009). In concordance with the observations in E11.5 embryos, *Dll1^lacZ/lacZ^* mesencephalic explants cultured for 2 or 4 days showed a higher proportion of Th+ cells in comparison with equivalent samples from *Dll1^+/+^* embryos. Most Th+ cells were NeuN+ in 2 days cultures of explants from embryos of either genotype, but fewer have this neuronal marker in 4 days cultures of *Dll1^lacZ/lacZ^* explants than of wild type (Fig. 5A). This is reminiscent to the process observed in newly born dopaminergic neurons of embryos at E13.5, which are NeuN+ and lost this marker by E15.5 (see Fig. 7). Therefore, late phases of dopaminergic neurogenesis in the absence of Dll1 can be observed in explant cultures, which showed more advanced differentiation/maturation with respect to that occurring in wild-type explants.

In order to get more insights into the direct role of Notch signalling in dopaminergic differentiation, we cultured mesencephalic explants in the presence or absence of DAPT, a potent γ-secretase inhibitor that blunts Notch activation at a key step after ligand binding (Crawford and Roelink, 2007). Due to the variations in number and distribution of differentiating cells in cultured explants, we analysed dopaminergic neurogenesis in explants by estimating mRNA levels of genes relevant to the process. As expected, the Notch inhibitor abolished *Hes5* expression in mesencephalic explants of E9.5 or E10.5 embryos after 2 days in culture, whereas *Hes1* expression was only partially affected (Fig. 5B), in close similarity with the observations in mutant embryos. In general, the effect of Notch signalling inhibition on mRNA levels of neuronal and dopaminergic genes was in agreement with the observations in *Dll1* deficient embryos (Fig. 5C). The proneurogenic effect of Notch inhibition was more pronounced in samples at E9.5 than at E10.5 (i.e. 4- vs 8-fold at either 2 days or 4 days treatment). Interestingly, there was no proportional decrease in the mRNA levels of *Nestin* (Fig. 5C), suggesting that a fraction of NPCs are dividing even under Notch signalling inhibition; nonetheless, note that Notch signalling inhibition reduced but did not affect the increasing rate of *Nestin* mRNA levels in E9.5 explants, suggesting that NPC differentiation but not proliferation is the target of the Notch signalling pathway. Therefore, Notch signalling inhibition reproduces the neurogenic effects of lacking Dll1, confirming that this ligand is the major mediator of Notch functions.

### Absence of Notch signalling modifies the flux of dopaminergic differentiation

**Fig. 6.**
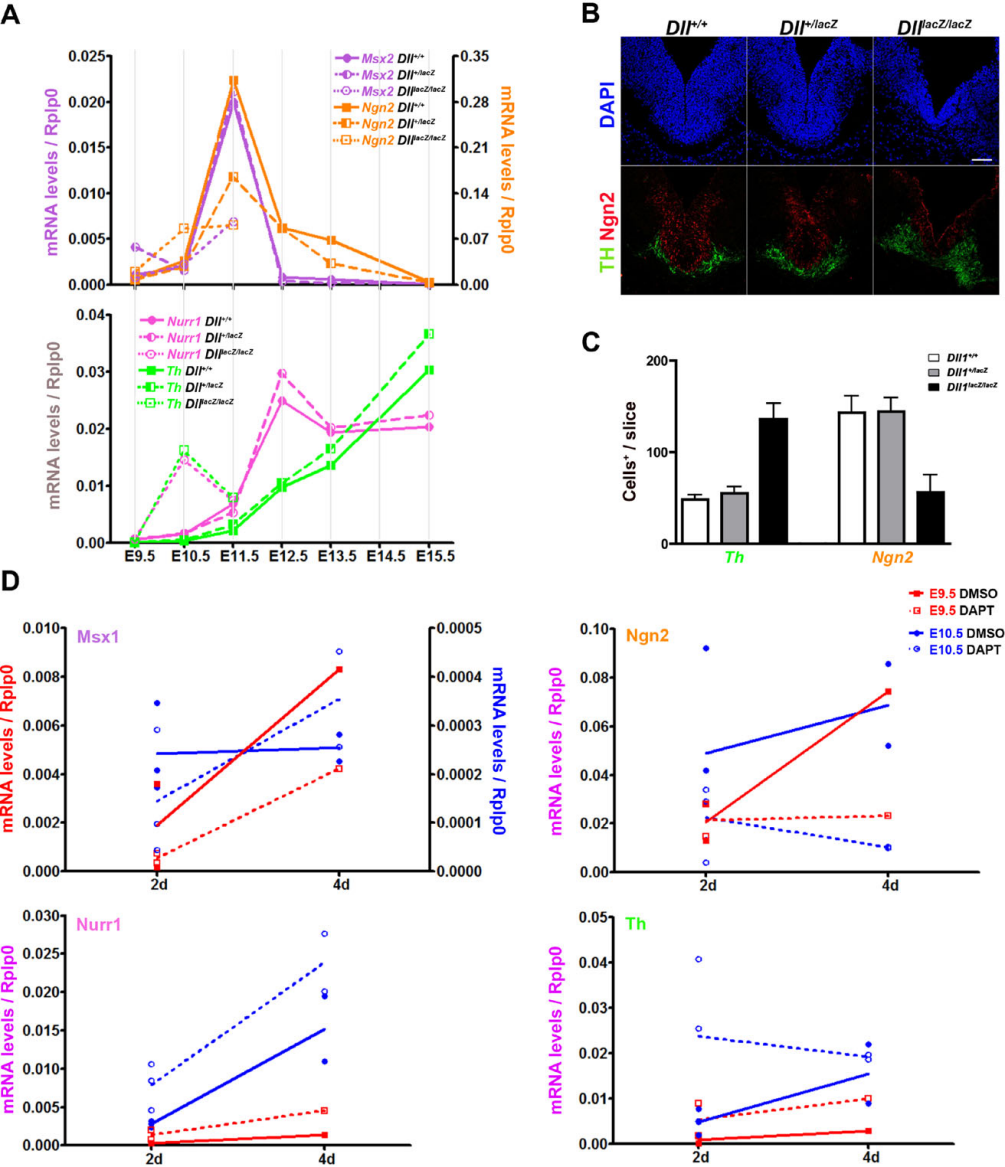
**Dopaminergic differentiation flux in mesencephalons along embryonic development or in explant cultures with normal or reduced Notch signalling.** Progression of dopaminergic differentiation was followed by detecting the expression of genes whose products are mainly associated with distinct dopaminergic NPCs (Msx1/2+, Ngn2+, Nurr1+) or differentiated neurons (*Th*). For developing mesencephalons (A), total RNA was extracted from a pool of ventral regions from embryos (at least 6) of the genotype indicated. For explant cultures of mesencephalons (D) from E9.5 or E10.5 embryos, treated or not (DMSO) with DAPT, each determination was from an independent pool of cultured explants (at least 3). Number of Ngn2+ NPCs or Th+ neurons in mesencephalons from E11.5 embryos with the genotype indicated (B,C) shows a good correlation with *Ngn2* mRNA levels. Scale bar is 100 µm. Data in C represented as average±s.d.

Msx1 and Ngn2 are transcription factors whose corresponding genes are transiently expressed during dopaminergic differentiation; the former is mostly present in ventricular NPCs and marks the initiation of dopaminergic differentiation (Andersson et al., 2006b), whereas the latter is the proneural factor more important for dopaminergic differentiation that shows higher levels in the intermediate progenitors before Nurr1 expression, a key transcription factor controlling *Th* expression (Kele et al., 2006). Because differentiating cells can spread to all directions from the site of birth, no direct relationship among the cells within a slice can be expected. Therefore, in order to estimate the transitions among the lineage-related dopaminergic precursors in the course of differentiation, we decided to determine the mRNA levels corresponding to those three transcription factors in ventral mesencephalons of embryos from E9.5 to E15.5 (Fig. 6A). The highest mRNA levels of *Msx1/2* were detected at E11.5, which represented a 30-fold increase in comparison with the level found at E9.5; this level value rapidly decreased to near the limit of detection since E12.5. Significant levels of *Ngn2* mRNA were detected at E10.5, became highest at E11.5, and markedly dropped by E15.5. In contrast, *Nurr1* mRNA levels increased gradually from E10.5 up to E12.5, stage at which apparently became stable. Since *Msx1/2*, *Ngn2* and *Nurr1* are expressed mostly in restricted non-overlapping NPCs that are linked along the dopaminergic differentiation pathway, the mRNA levels determined are in agreement with a differentiation flux starting with a definite number of Msx1+ NPCs that are converted into dopaminergic neurons passing sequentially through Ngn2+ and Nurr1+ NPCs. Note that the increase in *Th* mRNA levels, representing the young dopaminergic neuroblasts, lagged that of Nurr1 (see below).

The increase in *Th* mRNA levels and relative number of dopaminergic neurons in the absence of Dll1 suggest that Notch signalling regulates the emergence of dopaminergic features. A marked increase in *Ngn2*, *Nurr1* and *Th* mRNA levels in mesencephalon of E10.5 embryos lacking Dll1 (Fig. 6A) suggests that the cell populations expressing *Ngn2*, *Nurr1* and *Th* are prematurely emerging. Interestingly, at E11.5, *Msx2* and *Ngn2* mRNA levels in the ventral mesencephalon of *Dll1^lacZ/lacZ^* embryos were lower than in the one of wild-type embryos; *Msx1* and *Mash1* showed similar regulation as Msx2 and *Ngn2*, respectively (Fig. S4). In agreement with the correlation between the proportion of NPCs and mRNA levels, the decrease in *Ngn2* mRNA levels correlated with a decrease in the number of Ngn2+ NPCs (Fig. 6B,C). In contrast, *Nurr1* and *Pitx3*, two genes downstream *Ngn2* whose expression remains in the emerging Th+ neurons, showed slight differences in their mRNA levels in the presence or absence of Dll1 at E11.5; only *Pitx3* mRNA levels were in line with the increase in *Th* expression but in much lower proportion (Fig. 4). Of note was that the reduction in Dll1 (i.e. to the amount present in *Dll1^+/lacZ^* embryos) caused a marked drop in *Ngn2* mRNA levels only in embryos at E11.5 but produced similar mRNA levels and expression patterns as wild-type of all other genes tested (Fig. 6A). Therefore, reduction in Dll1 dose promotes dopaminergic differentiation in association with a decrement in the early specific NPCs (i.e. Msx2+ and Ngn2+).

The above observations suggest that Dll1-Notch signalling is controlling the differentiation flux once dopaminergic NPCs are specified such that, during the process, the effect is mainly noted in the reduction of Ngn2+ transient progenitors but not evident in the proportion of differentiated dopaminergic neuroblasts. The dopaminergic differentiation flux can be observed in explants cultures treated with DAPT (Fig. 6D). During culture of mesencephalic explants active differentiation was indicated by the rise in mRNA levels of *Msx1*, *Ngn2* and *Nurr1*. The differentiation dynamics in E9.5 and E10.5 explants was similar but the increase from 2 days to 4 days of culture in mRNA levels of early markers (i.e. *Msx1/2*, *Ngn2*) was less pronounced, whereas of late markers (i.e. *Nurr1*, *Th*) was more pronounced at the more advanced developmental stage, consistent with the differentiation trend occurring. Upon Notch signalling inhibition, consistent decrease in *Msx1* mRNA levels was observed in E9.5 explants despite their increasing phase at this developmental stage which was still observed; this observation is in agreement with a no relevant function of Notch in dopaminergic NPC proliferation. In contrast, Notch inhibition caused a consistent decrease in *Ngn2* mRNA levels in E10.5 explants, whereas this effect was noted in E9.5 only after 4 days DAPT inhibitor treatment; apparent lack of effect on *Ngn2* mRNA levels in 2 days-treated E9.5 explants supports a transitory role of Ngn2+ cells during differentiation. *Nurr1* mRNA levels showed lower levels in E9.5 than in E10.5 explants, but a higher increment was triggered by Notch inhibition. Similar behaviour was observed for the *Th* mRNA but its levels did not rise in E10.5 explants after 4 days Notch inhibitor treatment possibly due to the depletion of progenitor cells. Together, these data are in agreement with a model in which the proliferation and size of the pool of NPCs (i.e. E9.5>E10.5) defines how the dopaminergic differentiation flux is affected by Notch inhibition such that, NPC depletion and neuron generation induced by the Notch inhibitor strengthen and weaken, respectively, at late phases of differentiation (possibly after E11.5).

### *Dll1* haploinsufficiency causes a reduction in dopaminergic neurons of the adult brain substantia nigra

**Fig. 7.**
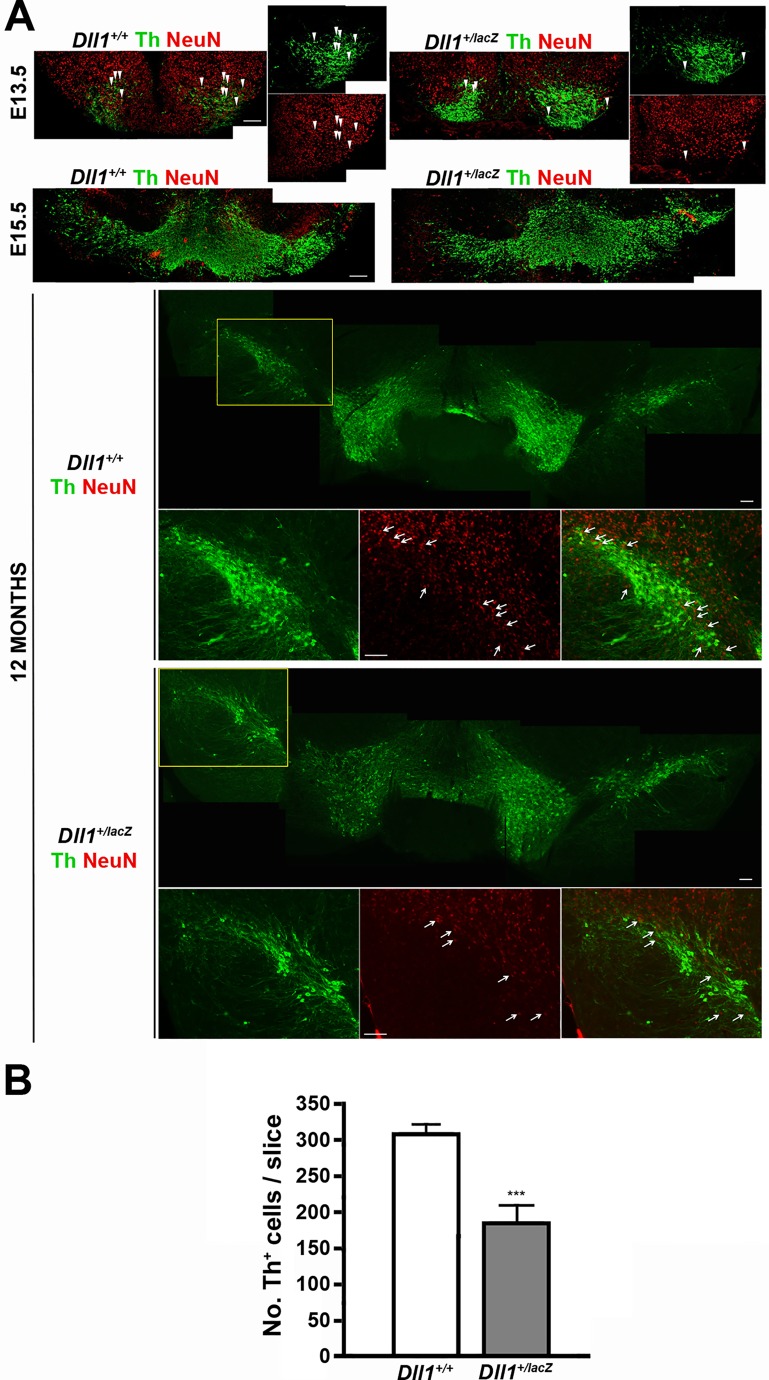
**Number of dopaminergic neurons in the adult brain under reduced Dll1 levels.** (A) Dopaminergic neurons were detected by the presence of Th (green). No apparent difference in the density of Th+ neurons was detected in embryonic samples. Note that some Th+ neurons in the mesencephalon of E13.5 embryos (arrowheads) but not in that of E15.5 embryos contained nuclear NeuN (red), a marker frequently found in mature neurons. Evident lower density of Th+ neurons, all lacking nuclear NeuN, was observed in the SNpc of *Dll^+/lacZ^* mice in comparison with the density in this region of wild-type mice. Scale bars are 100 µm; arrows indicate Th−/NeuN+ cells within the SNpc. (B) Th+ neurons were counted in slices from brains of 14 month-old mice. Data represented as average±s.d.

The alterations in mRNA levels of genes involved in neuronal dopaminergic differentiation as well as the mesencephalic phenotype observed in *Dll1^+/lacZ^* embryos, both consistent with mild premature neuronal differentiation, prompted us to study the consequences in adult mice. Although no differences in mRNA levels of neuronal or dopaminergic differentiation markers were detected at E13.5 or E15.5, lower density of dopaminergic neurons in the ventral mesencephalon of mutant in comparison with wild-type mice was not obvious at E13.5 but apparent at E15.5 when they are nearly to establish their final allocation (Fig. 7A). As mentioned above, NeuN downregulation correlated with dopaminergic neuron maturation such that some Th+/NeuN+ were detected in *Dll1^+/lacZ^* E13.5 embryos but almost none in E15.5 embryos of either genotype. Interestingly, this reduction in dopaminergic neurons became evident in adult mice, where it was readily visible in the substantia nigra pars compacta (SNpc) where most Th+ neurons remained NeuN negative (Fig. 7A,B). This is not related to a Dll1 role in neuron survival as similar phenotype was observed in young and old mice (see Fig. S5). Nonetheless, lower neuronal density was not restricted to dopaminergic neurons or the mesencephalic area but rather noted throughout the brain. The functional consequences of this reduction in neuronal density will be published elsewhere (manuscript in preparation). Therefore, a lower dose of Dll1 decreases the production of several neuronal types, possibly by disrupting a differentiation flux similar to the one described for mesencephalic dopaminergic neurons.

### A mathematical model of dopaminergic differentiation flux

**Fig. 8.**
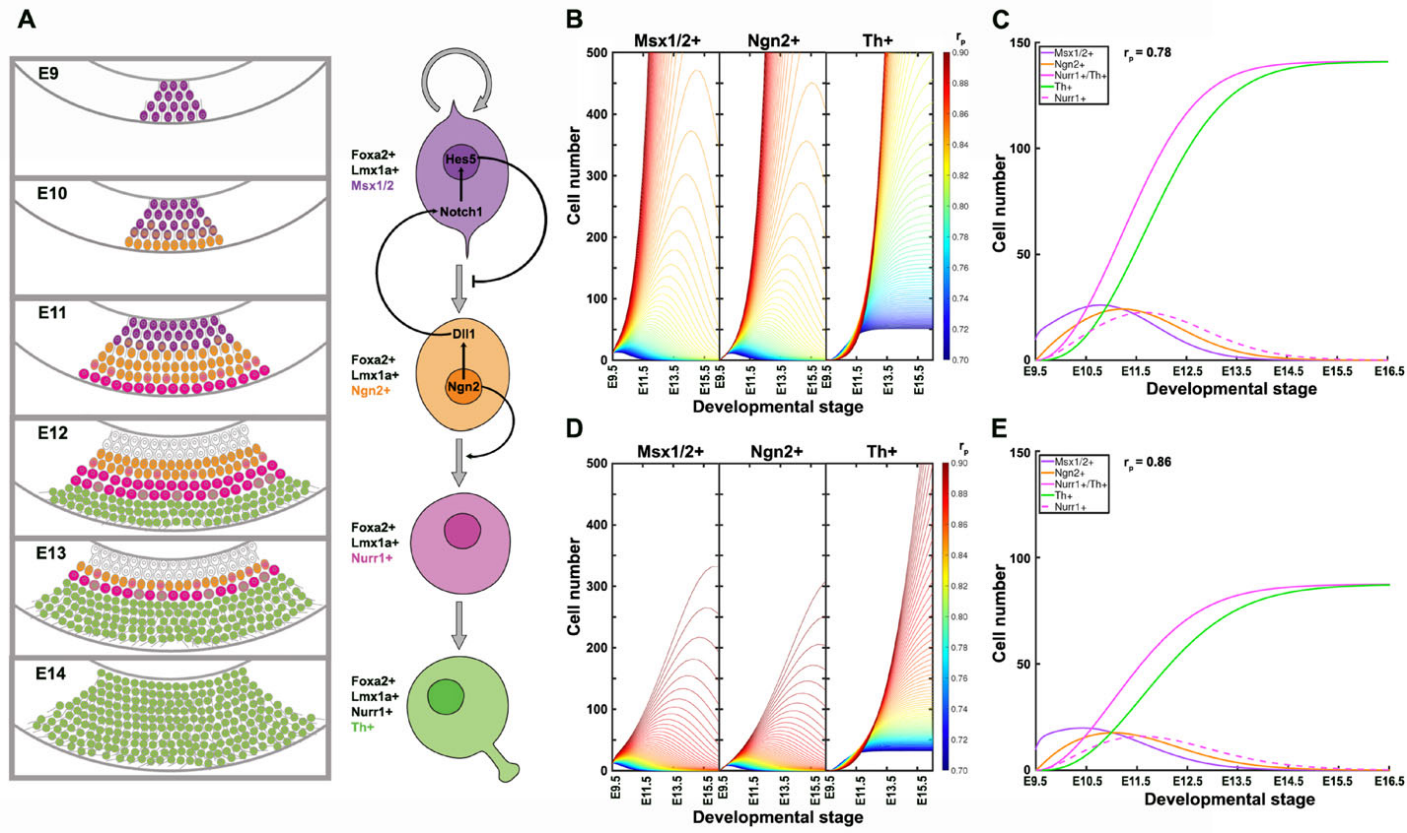
**A differentiation flux mathematical model for the derivation of mesencephalic dopaminergic neurons.** (A) Representations of the distinct cell populations analysed during dopaminergic differentiation in the ventral mesencephalon and of the cell differentiation progress indicating the proposed role of Dll1 and Ngn2. (B,D) Dynamics of distinct cell populations (Msx1/2+, Ngn2+, Th+) emerging during dopaminergic differentiation at different r_p_ values in the presence of the complete (full dose of Dll1; B) or half (e.g. that present in *Dll^+/lacZ^*; D) active I_1_ population. (C,E) The dynamic growth of the major NPC populations and immature dopaminergic neurons at a selected x and r_p_ values, those giving rise to patterns resembling the ones experimentally determined. For the dynamics shown, P_0_=10.

During neurogenesis, early ventricular neural precursors move to the subventricular zone as they differentiate (Fig. 8A). *Dll1* expression was detected in the subventricular area of most mesencephalon at E10-E11 (Fig. 1) (see also Kele et al., 2006; Lahti et al., 2011; Ono et al., 2007), whereas Hes5, the main mediator of Dll1-Notch signalling found, has been reported to be mostly located in the ventricular area (Kele et al., 2006; Vernay et al., 2005). From these observations we inferred that cells targeted by Dll1 are located in the ventricular zone (Fig. 8A), though we cannot discard a partial overlapping between *Dll1* and *Hes5* expression domains. Analysis of cell death and proliferation and emergence of neuronal markers at different amounts of Dll1 (i.e. those in *Dll1^+/+^*, *Dll1^+/LacZ^*, *Dll1^lacZ/lacZ^* mice) suggest that as Dll1 decreases, the differentiation rate increases, meaning that fewer NPCs divide and more exit the cell cycle to become neuroblasts. This phenomenon has been observed in other brain regions and interpreted as premature neuronal differentiation due to the lack of the inhibitory differentiation effect of Notch signalling (Hatakeyama et al., 2004; de la Pompa et al., 1997; Louvi and Artavanis-Tsakonas, 2006). Premature differentiation during the expansion of dividing precursors could cause their rapid exhaustion and, consequently, a reduction in the number of neurons produced.

Increased rate of differentiation can also be observed following specifically the mesencephalic dopaminergic lineage (Fig. 8A). Our data show that initiation of neuronal differentiation, as detected by the emergence of βIII-tubulin, is closely coupled with the acquisition of markers of early dopaminergic neuroblasts such as Nurr1 and Th, and negatively regulated by Dll1. As expected, Msx1/2, a marker of early dividing dopaminergic precursors, concomitantly decreases. As inferred from mRNA levels, the number of Ngn2+ cells increases and decreases in coordination with that of Msx1/2+ cells, but under reduced Dll1 levels, early emergence of Ngn2+ cells occurs (Fig. 6A). Because the Ngn2+ NPCs population is a transient poorly dividing cell population (Andersson et al., 2006a; Kele et al., 2006; Thompson et al., 2006), these observations support a mechanism in which there is no additional restriction on differentiation from Ngn2+ progenitors into Nurr1+ neuroblasts.

A simplified model to describe the transition from specified dopaminergic precursor (Msx1/2+; P), going through intermediate progenitors (Ngn2+, Nurr1+; I) up to becoming a young dopaminergic neuron (Nurr1+/Th+; N) (Fig. 8A) is: 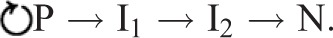
Given that Ngn2 controls *Dll1* expression, effects of Dll1 on P are proportional to the amount of I adjacent to P (I_1_; I_2_ refers to the I population moving away from P possibly Nurr1+), thus, this effect varies according with the equation
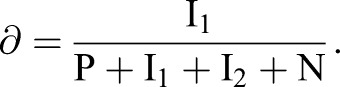
Considering that there is no restriction on differentiation from I to N, as data suggested, the dynamic change in the number of P, I_1_, I_2_ and N during differentiation can be modelled by the following set of differential equations,1
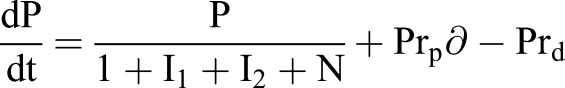
2
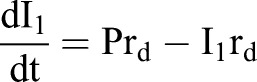
3
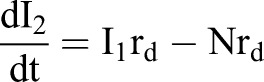
4
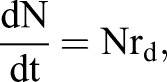
which develop over time from an initial population P_0_ (P when I_1_+I_2_+N=0) and where the term P/(1+I_1_+I_2_+N) represents the fraction of P with proliferation independent of Dll1-Notch signalling. r_p_ and r_d_ denote the probability that one P cell self-divides or differentiates, respectively. Thus,
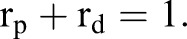
To estimate the value of r_p_ and r_d_, we consider our experimental observations showing that the population Msx1/2+ (P) initially grows and extinguishes during the 6 days of differentiation (E9-E15) with a maximum around E11.5, and that the plateau of dopaminergic neurons (Th+; N) is reached between E13.5-E14.5. Using Eqns 1-4 and P_0_=10 to describe the differentiation dynamics, it can be determined that P grows when the r_p_ value is above 0.70, and gets restricted to the short range of 0.75-0.80 when considering the time at which N reaches a plateau (Fig. 8B). The r_p_/r_d_ value might correlate with a short window of the cell cycle at which the neurogenic process can initiate (Latasa et al., 2009).

Fig. 8C shows the dynamics of dopaminergic differentiation by comparing the growth pattern of the major distinct cell populations considered in this work (P_0_=10, r_p_=0.78). Interestingly, the pattern shown resembles the one experimentally determined using the gene expression levels of the specific markers for P, I_1_, I_2_ and N (Fig. 6). A major conclusion derived from this dynamic model is that, the initial P_0_ population produces a 15-20-fold larger N population, which implies that P divides every ∼20 h 4-5 times during differentiation. Also, it can be inferred that the Nurr1 gene expression pattern determined is associated with a transient Nurr1+/Th− population (possibly I_2_ in the model) that emerges before becoming Nurr1+/Th+ neuroblasts (N).

In the simple mathematical model described above, ∂=0 represents complete Dll1 deficiency (no Notch signalling), and ∂_x_=I_1_(x)/[P+I_1_(x)+I_2_+N] when there is ‘x’ fraction of I_1_ than in wild-type embryos, an estimation of the Dll1 level triggering Notch signalling in P; for instance, x in *Dll1* heterozygous embryos might equal ½. Varying ∂ values in the above equations give rise to the dynamics such as the ones represented in Fig. 8D (x=0.5; see also Fig. S6 for x=0) showing that, as expected, earlier emergence and lower production of dopaminergic neurons than wild-type occur as Notch signalling decreases. Note, however, that the production of dopaminergic neurons at the same r_p_ value used when x=1 (i.e. full Dll1 dose) appears much lower than the one experimentally determined in the SNpc of *Dll1^+/LacZ^* (about 80% of wild type). Considering that the Dll1 dose in heterozygous mice is actually half that of wild type, we propose that compensatory mechanisms controlling differentiation under low Dll1 dose are reflected in small changes in the r_p_ value (Fig. 8E). Therefore, dopaminergic differentiation dynamics is markedly influenced by the regulatory mechanisms acting on P to precisely determine the level of proliferation and differentiation.

### Concluding remarks

The initial specification of mesencephalic dopaminergic NPCs occurs in the absence of Notch signalling. This is not unexpected since *Dll1* expression in the floor plate depends on a functional *Ngn2*, a gene downstream *Foxa2* and *Lmx1a* (Andersson et al., 2006b; Ferri et al., 2007). A very similar phenomenon occurs in the spinal cord where homeodomain proteins define the Dll1 spatial pattern and lack of Dll1 increases neuronal differentiation without affecting the specific progenitor domain boundaries (Marklund et al., 2010). Ngn2 marks a transient NPC population during dopaminergic differentiation, where positively regulates *Dll1* expression (Castro et al., 2006; Kele et al., 2006), while the data presented here show that Dll1 does not appear to regulate the expression of *Ngn2* but rather determines the number of cells expressing it (Fig. 8A). Midbrains of *Ngn2^−/−^* embryos show a marked reduction in Ngn2 downstream dopaminergic markers such as Nurr1, Pitx3 and Th (Kele et al., 2006) that, after birth, results in a significant decrease in the number of dopaminergic neurons (Andersson et al., 2006a). Considering our observations, we propose that, in addition to the neurogenic role, Ngn2 has an additional unique function in dopaminergic differentiation, the regulation of NPC expansion through Dll1 (Fig. 8A). Nonetheless, additional mechanisms compensate the effect of reducing Ngn2 (Andersson et al., 2006a; Kele et al., 2006) or Dll1 (this work) on the production of dopaminergic neurons. The dynamic model of differentiation we propose indicate that dopaminergic NPCs exhaust due to a higher rate of differentiation than proliferation, a process regulated by the interaction between Dll1 and Ngn2. The remaining NPCs after E12.5 are likely the source of the glial cells emerging later in development.

The production of specific neurons during development should comply with the number needed for their functions in the adult brain. Moreover, it is expected that neuronal differentiation is coordinated in time with the processes that control migration of neuroblasts or young neurons to the definitive residence site, such that successful connections between neurons occur. It is generally thought that more neurons than needed are produced and that many die as they move through the path to their home and their axons compete for reaching the target cells. However, only in few instances have been demonstrated the role of natural neurodegeneration in the establishment of the final number of neurons, therefore, it is still relevant to determine the contribution of neurons produced. Here we found that the Notch-regulated differentiation flux from mesencephalic dopaminergic precursors up to becoming dopaminergic neurons is critical for determining the number of dopaminergic neurons present in the adult brain.

## MATERIALS AND METHODS

### Maintenance of mutant mouse line *DII1^lacZ/^*^+^

The *DII1^lacZ/+^* mutant mouse line in the CD1 strain genetic background was kindly provided by Dr Olivier Pourquié at the Stowers Institute for Medical Research. Because CD1 is an outbred strain, phenotypes resulting from the *Dll1* mutation or its penetrance for certain phenotypes might vary among homozygous embryos; nonetheless, the particular phenotype studied here was highly reproducible suggesting low influence of potential modifiers present in this strain. The mutation resulted from the in frame replacement of exon1 by the *lacZ* gene, as described by Hrabĕ de Angelis et al. (1997). The null allele was maintained in the heterozygous state, since homozygous *DII1^lacZ/lacZ^* embryos die at around embryonic day E12.5, similar as previously reported (Hrabĕ de Angelis et al., 1997). With little variation, the genotype determined by PCR (forward primer: 5′-GTTGCACCACAGATGAAACGC-3′; reverse primer: 5′-AAGCCAGACGAAGAGGAAACCG-3′) was coincident with *lacZ* expression level and with E10-E12 embryo phenotype (e.g. head morphology, haemorrhagic spots). All animal manipulations required for the present work were in compliance with the ‘Guide for the Care and Use of Laboratory Animals’ (National Research Council) and approved by our Bioethical Committee.

### Collagen explant cultures

The midbrain explants were prepared as described by Baizabal and Covarrubias (2009). When explants (E9.5 or E10.5) were treated with the γ-secretase inhibitor *N*-[(3,5-Difluorophenyl)acetyl]-L-alanyl-2-phenyl]glycine-1,1-dimethylethyl ester (DAPT) (5 µM, Tocris), control medium had same volume (0.5 µl) of dimethylsulfoxide (DMSO); under these conditions, explants were not cultured for longer than 4 days.

### Tissue processing and immunofluorescence

Embryonic brain tissue and midbrain explants sections were processed as described by Baizabal and Covarrubias (2009). All coronal sections analysed were perpendicular to the tangent near the midpoint of the mesencephalic flexure and those showing the complete ventral neuroepithelium (Fig. S1); thus, the extreme anterior and posterior mesencephalic areas were excluded. Tissue sections were incubated with the appropriate mixture of antibodies (Table S1). To determine active cell proliferation, pregnant mice were injected with 50 µl of BrdU (50 µg/µl; Sigma-Aldrich) 1 h before sacrifice; BrdU incorporation was determined by immunofluorescence. On the other hand, the TUNEL assay (Roche) was used to detect apoptotic cells in embryo brain cryosections. Preparations were counterstained with DAPI (1:10,000; Invitrogen), and analysed for immunofluorescence using a Zeiss LSM 510 confocal microscope and Zeiss Apotome microscope Axio Observer Z1.

### Real-time quantitative RT-PCR

Total RNA was extracted with TRIzol (Invitrogen) following the manufacturer's instructions. The ventral midbrain (defined by the one-forth of midbrain around the midline) of embryos was dissected and pooled for RNA extraction from no less than two litters at each developmental stage tested (i.e. E9.5-E15.5 dpc). Single ventral midbrains were not used for this analysis because rapid changes in gene expression occur in this region within the relevant developmental window, and because the low RNA yield would prevent from using the same sample for comparing the expression pattern of all genes. For explants, 3 midbrain explants (E9.5 or E10.5) of each condition and/or culture stage were collected per experiment; at least two experiments were performed per condition and/or culture stage. First strand cDNA was synthetized using AMV Reverse Transcriptase (Invitrogen) and oligo-dT_16_ primer. Quantitative RT-PCR was performed using KAPA SYBR FAST mix (KAPA Biosystems) in the presence of the specific primers (Table S2) and the Rotor-Gene 3000 thermocycler (Corbett Research). Gene expression was evaluated using a ΔΔC_t_ method. The housekeeping gene *Rplp0* was used to normalize gene expression levels.

### Data analysis and mathematical modelling

For cell quantification in tissue sections of embryos and explants cultures, single optical planes were analysed independently within each confocal stack. Total number of cells positive for the specific marker was determined within the stack. Unless indicated, cells counted were within the marked area, usually corresponding to the Lmx1a expression domain (determined in a parallel section). For adult brains, mice of 5, 8, 12 and 14 months of age were used. Although difference between *DII1^+/+^* and *DII1^lacZ/lacZ^* in the number of Th+ neurons was similar at all ages, robust analysis was done for one-year old animals. We counted the number of Th+ cells in the SNpc of five different slices along the antero-posterior axis for each *DII1^+/+^* and *DII1^lacZ/lacZ^* mouse (*n*=4). The proportion of positive cells for a given marker is expressed as a percentage of the total cell number (DAPI+ cells)±s.d. (standard deviation) calculated from at least 3 different tissue samples. Asterisks in graphs indicate that the experimental groups were significantly different from control groups (*P*<0.05 as determined by the *t*-test). Mathematical modelling was performed in Matlab softaware (MathWorks, Natick, Massachusetts).

## References

[BIO013383C1] Abeliovich A. and Hammond R. (2007). Midbrain dopamine neuron differentiation: factors and fates. *Dev. Biol.* 304, 447-454. 10.1016/j.ydbio.2007.01.032 17331494

[BIO013383C2] Andersson E., Jensen J. B., Parmar M., Guillemot F. and Björklund A. (2006a). Development of the mesencephalic dopaminergic neuron system is compromised in the absence of neurogenin 2. *Development* 133, 507-516. 10.1242/dev.02224 16396906

[BIO013383C3] Andersson E., Tryggvason U., Deng Q., Friling S., Alekseenko Z., Robert B., Perlmann T. and Ericson J. (2006b). Identification of intrinsic determinants of midbrain dopamine neurons. *Cell* 124, 393-405. 10.1016/j.cell.2005.10.037 16439212

[BIO013383C4] Ang S.-L. (2006). Transcriptional control of midbrain dopaminergic neuron development. *Development* 133, 3499-3506. 10.1242/dev.02501 16899537

[BIO013383C5] Baizabal J.-M. and Covarrubias L. (2009). The embryonic midbrain directs neuronal specification of embryonic stem cells at early stages of differentiation. *Dev. Biol.* 325, 49-59. 10.1016/j.ydbio.2008.09.024 18929554

[BIO013383C6] Bassett E. A. and Wallace V. A. (2012). Cell fate determination in the vertebrate retina. *Trends Neurosci.* 35, 565-573. 10.1016/j.tins.2012.05.004 22704732

[BIO013383C116] Bettenhausen B. and Gossler A. (1995). Efficient isolation of novel mouse genes differentially expressed in early postimplantation embryos. *Genomics.* 28, 436-441. 10.1006/geno.1995.1172 7490078

[BIO013383C7] Castro D. S., Skowronska-Krawczyk D., Armant O., Donaldson I. J., Parras C., Hunt C., Critchley J. A., Nguyen L., Gossler A. and Göttgens B. (2006). Proneural bHLH and Brn proteins coregulate a neurogenic program through cooperative binding to a conserved DNA motif. *Dev. Cell* 11, 831-844. 10.1016/j.devcel.2006.10.006 17141158

[BIO013383C8] Conlon R. A., Reaume A. G. and Rossant J. (1995). Notch1 is required for the coordinate segmentation of somites. *Development* 121, 1533-1545. 7789282 10.1242/dev.121.5.1533

[BIO013383C9] Crawford T. Q. and Roelink H. (2007). The notch response inhibitor DAPT enhances neuronal differentiation in embryonic stem cell-derived embryoid bodies independently of sonic hedgehog signaling. *Dev. Dyn.* 236, 886-892. 10.1002/dvdy.21083 17295317

[BIO013383C10] de la Pompa J. L., Wakeham A., Correia K. M., Samper E., Brown S., Aguilera R. J., Nakano T., Honjo T., Mak T. W., Rossant J. et al. (1997). Conservation of the Notch signalling pathway in mammalian neurogenesis. *Development* 124, 1139-1148. 9102301 10.1242/dev.124.6.1139

[BIO013383C11] Deng Q., Andersson E., Hedlund E., Alekseenko Z., Coppola E., Panman L., Millonig J. H., Brunet J.-F., Ericson J. and Perlmann T. (2011). Specific and integrated roles of Lmx1a, Lmx1b and Phox2a in ventral midbrain development. *Development* 138, 3399-3408. 10.1242/dev.065482 21752929

[BIO013383C12] Duarte A., Hirashima M., Benedito R., Trindade A., Diniz P., Bekman E., Costa L., Henrique D. and Rossant J. (2004). Dosage-sensitive requirement for mouse Dll4 in artery development. *Genes Dev.* 18, 2474-2478. 10.1101/gad.1239004 15466159 PMC529534

[BIO013383C13] Ferri A. L. M., Lin W., Mavromatakis Y. E., Wang J. C., Sasaki H., Whitsett J. A. and Ang S.-L. (2007). Foxa1 and Foxa2 regulate multiple phases of midbrain dopaminergic neuron development in a dosage-dependent manner. *Development* 134, 2761-2769. 10.1242/dev.000141 17596284

[BIO013383C14] Guerrero-Flores G. and Covarrubias L. (2011). Dopaminergic differentiation potential of neural precursor cells derived from embryonic stem cells. In *Embryonic Stem Cells: The Hormonal Regulation of Pluripotency and Embryogenesis* (ed. Atwood C.), pp. 413-428. Rijeka, Croatia: InTech.

[BIO013383C15] Hatakeyama J., Bessho Y., Katoh K., Ookawara S., Fujioka M., Guillemot F. and Kageyama R. (2004). Hes genes regulate size, shape and histogenesis of the nervous system by control of the timing of neural stem cell differentiation. *Development* 131, 5539-5550. 10.1242/dev.01436 15496443

[BIO013383C16] Hegarty S. V., Sullivan A. M. and O'Keeffe G. W. (2013). Midbrain dopaminergic neurons: a review of the molecular circuitry that regulates their development. *Dev. Biol.* 379, 123-138. 10.1016/j.ydbio.2013.04.014 23603197

[BIO013383C17] Hrabĕ de Angelis M., McIntyre J. and Gossler A. (1997). Maintenance of somite borders in mice requires the Delta homologue DII1. *Nature* 386, 717-721. 10.1038/386717a0 9109488

[BIO013383C311] Joksimovic M., Yun B. A., Kittappa R., Anderegg A. M., Chang W. W., Taketo M. M., McKay R. D. G. and Awatramani R. B. (2009). Wnt antagonism of Shh facilitates midbrain floor plate neurogenesis. *Nat. Neurosci.* 12, 125-131. 10.1038/nn.2243 19122665

[BIO013383C18] Kadokawa Y. and Marunouchi T. (2002). Chimeric analysis of Notch2 function: a role for Notch2 in the development of the roof plate of the mouse brain. *Dev. Dyn.* 225, 126-134. 10.1002/dvdy.10140 12242712

[BIO013383C19] Kele J., Simplicio N., Ferri A. L. M., Mira H., Guillemot F., Arenas E. and Ang S.-L. (2006). Neurogenin 2 is required for the development of ventral midbrain dopaminergic neurons. *Development* 133, 495-505. 10.1242/dev.02223 16410412

[BIO013383C20] Kittappa R., Chang W. W., Awatramani R. B. and Mckay R. D. G. (2007). The foxa2 gene controls the birth and spontaneous degeneration of dopamine neurons in old age. *PLoS Biol.* 5, e325 10.1371/journal.pbio.0050325 18076286 PMC2121110

[BIO013383C21] Lahti L., Saarimäki-Vire J., Rita H. and Partanen J. (2011). FGF signaling gradient maintains symmetrical proliferative divisions of midbrain neuronal progenitors. *Dev. Biol.* 349, 270-282. 10.1016/j.ydbio.2010.11.008 21074523

[BIO013383C22] Latasa M. J., Cisneros E. and Frade J. M. (2009). Cell cycle control of Notch signaling and the functional regionalization of the neuroepithelium during vertebrate neurogenesis. *Int. J. Dev. Biol.* 53, 895-908. 10.1387/ijdb.082721ml 19598111

[BIO013383C23] Lin W., Metzakopian E., Mavromatakis Y. E., Gao N., Balaskas N., Sasaki H., Briscoe J., Whitsett J. A., Goulding M., Kaestner K. H. et al. (2009). Foxa1 and Foxa2 function both upstream of and cooperatively with Lmx1a and Lmx1b in a feedforward loop promoting mesodiencephalic dopaminergic neuron development. *Dev. Biol.* 333, 386-396. 10.1016/j.ydbio.2009.07.006 19607821

[BIO013383C24] Lindsell C. E., Boulter J., diSibio G., Gossler A. and Weinmaster G. (1996). Expression patterns of Jagged, Delta1, Notch1, Notch2, and Notch3 genes identify ligand-receptor pairs that may function in neural development. *Mol. Cell. Neurosci.* 8, 14-27. 10.1006/mcne.1996.0040 8923452

[BIO013383C25] Louvi A. and Artavanis-Tsakonas S. (2006). Notch signalling in vertebrate neural development. *Nat. Rev. Neurosci.* 7, 93-102. 10.1038/nrn1847 16429119

[BIO013383C26] Lütolf S., Radtke F., Aguet M., Suter U. and Taylor V. (2002). Notch1 is required for neuronal and glial differentiation in the cerebellum. *Development* 129, 373-385. 11807030 10.1242/dev.129.2.373

[BIO013383C27] Marklund U., Hansson E. M., Sundström E., de Angelis M. H., Przemeck G. K. H., Lendahl U., Muhr J. and Ericson J. (2010). Domain-specific control of neurogenesis achieved through patterned regulation of Notch ligand expression. *Development* 137, 437-445. 10.1242/dev.036806 20081190

[BIO013383C28] Nakatani T., Kumai M., Mizuhara E., Minaki Y. and Ono Y. (2010). Lmx1a and Lmx1b cooperate with Foxa2 to coordinate the specification of dopaminergic neurons and control of floor plate cell differentiation in the developing mesencephalon. *Dev. Biol.* 339, 101-113. 10.1016/j.ydbio.2009.12.017 20035737

[BIO013383C29] Ohtsuka T., Ishibashi M., Gradwohl G., Nakanishi S., Guillemot F. and Kageyama R. (1999). Hes1 and Hes5 as notch effectors in mammalian neuronal differentiation. *EMBO J.* 18, 2196-2207. 10.1093/emboj/18.8.2196 10205173 PMC1171303

[BIO013383C30] Okano H. and Temple S. (2009). Cell types to order: temporal specification of CNS stem cells. *Curr. Opin. Neurobiol.* 19, 112-119. 10.1016/j.conb.2009.04.003 19427192

[BIO013383C31] Ono Y., Nakatani T., Sakamoto Y., Mizuhara E., Minaki Y., Kumai M., Hamaguchi A., Nishimura M., Inoue Y., Hayashi H. et al. (2007). Differences in neurogenic potential in floor plate cells along an anteroposterior location: midbrain dopaminergic neurons originate from mesencephalic floor plate cells. *Development* 134, 3213-3225. 10.1242/dev.02879 17670789

[BIO013383C32] Pierfelice T., Alberí L. and Gaiano N. (2011). Notch in the vertebrate nervous system: an old dog with new tricks. *Neuron* 69, 840-855. 10.1016/j.neuron.2011.02.031 21382546

[BIO013383C33] Shen Q., Wang Y., Dimos J. T., Fasano C. A., Phoenix T. N., Lemischka I. R., Ivanova N. B., Stifani S., Morrisey E. E. and Temple S. (2006). The timing of cortical neurogenesis is encoded within lineages of individual progenitor cells. *Nat. Neurosci.* 9, 743-751. 10.1038/nn1694 16680166

[BIO013383C34] Swiatek P. J., Lindsell C. E., del Amo F. F., Weinmaster G. and Gridley T. (1994). Notch1 is essential for postimplantation development in mice. *Genes Dev.* 8, 707-719. 10.1101/gad.8.6.707 7926761

[BIO013383C35] Thompson L. H., Andersson E., Jensen J. B., Barraud P., Guillemot F., Parmar M. and Björklund A. (2006). Neurogenin2 identifies a transplantable dopamine neuron precursor in the developing ventral mesencephalon. *Exp. Neurol.* 198, 183-198. 10.1016/j.expneurol.2005.11.025 16438966

[BIO013383C36] Vernay B., Koch M., Vaccarino F., Briscoe J., Simeone A., Kageyama R. and Ang S.-L. (2005). Otx2 regulates subtype specification and neurogenesis in the midbrain. *J. Neurosci.* 25, 4856-4867. 10.1523/JNEUROSCI.5158-04.2005 15888661 PMC6724764

